# Profiling of CSF: Author's Reply

**DOI:** 10.1371/journal.pmed.0030468

**Published:** 2006-10-31

**Authors:** Sabine Bahn, F. Markus Leweke, Jeffrey T.-J Huang

We are grateful to Dr. Matthews for his comments [1]. With a sensitivity of 82% and a specificity of 85%, the metabolic markers for schizophrenia identified in our study are certainly not perfect. However, we have already identified further biomarkers and envisage that a panel of biomarkers can be used for diagnostic purposes. The utility of these biomarkers will be established in our future clinical studies.

We take the point of Dr. Hambridge [2], and agree that patients who present with schizophrenia-like symptoms need to be fully investigated with respect to possible (treatable) underlying pathologies. However, we strongly disagree with the concept of schizophrenia not being an “organic disorder”. Unfortunately, as Dr. Hambridge points out, this atavistiv view is still portrayed in the current classification systems, but we hope that by now most psychiatrists would agree that schizophrenia has a biological/organic aetiology. The concept of schizophrenia as a “functional psychosis” is a misnomer, as every function has a biological/organic basis.

As stated in our article, all patients included in our study underwent a thorough neurological and psychiatric examination. The majority of patients had cMRI scans or cCT scans (when MRI was not available at the time of admission), and a battery of blood tests including syphilis and endocrinological screening. Serum and CSF testing for neurotropic viruses and borreliosis as well as routine parameters were undertaken in accordance with European guidelines for cerebrospinal fluid (CSF) diagnostics.

Furthermore, patients had urine drug screens, an electroencephalogram, and were examined using a neuropsychological test-battery as well as having an optional HIV test as first-line investigation. For our study as well as for our daily clinical routine, the diagnosis of schizophrenia or schizophreniform disorder (with regard to time criteria only) is only given if no findings indicative of other neuropsychiatric/neurological disorders, other than schizophrenia, were obtained. We would like to emphasise that it is not our opinion that the possible use of biomarker profi les, such as those identifi ed in our study, would eliminate the need for extensive neurological/biochemical screening of any patient with psychosis. Instead, our study strongly justifi es the need to perform an extensive battery of clinical tests. Unfortunately, this is not clinical practice in many countries, not least for reasons of cost and the low profile that psychiatric patients have within our health systems.
